# Ischemic Monomelic Neuropathy after Arteriovenous Fistula Surgery: Clinical Features, Electrodiagnostic Findings, and Treatment

**DOI:** 10.7759/cureus.5191

**Published:** 2019-07-22

**Authors:** Sorabh Datta, Shanan Mahal, Raghav Govindarajan

**Affiliations:** 1 Neurology, University of Missouri, Columbia, USA; 2 Internal Medicine, Baptist Health-University of Arkansas for Medical Sciences, North Little Rock, USA

**Keywords:** hemodialysis, dialysis access associated steal syndrome (dass), diabetes, av fistula graft, emg/ncs, ischemia, esrd (end stage renal disease), sensorimotor neuropathy, post surgical pain, neuro-regeneration

## Abstract

Ischemic monomelic neuropathy (IMN) is a rare complication of vascular access in the hemodialysis patients, characterized by multiple mononeuropathies in the absence of clinical ischemia. Most commonly seen in the female gender, diabetes mellitus, and it must be differentiated from vascular steal syndrome, where we see clinical ischemia as the main pathognomonic feature. Early recognition of the symptoms and prompt intervention was shown to be beneficial. A delay in the treatment can lead to irreversible damage to the nerves and muscles. This article is depicting a case of an elderly male patient who presented with signs and symptoms of IMN which developed after arteriovenous (AV) fistula graft surgery.

## Introduction

Ischemic monomelic neuropathy (IMN) is one of the rare complication encountered after arteriovenous (AV) fistula graft surgery. As the name suggests, ischemia or the impaired blood supply is the main pathognomonic reason for ischemic monomelic neuropathy [1]. IMN is characterized by symptoms of acute pain, numbness, paresthesia along with the motor weakness, and it is most likely to occur in patients with brachiocephalic vascular graft [2]. Here we are presenting a case of IMN in a patient with ESRD (end-stage renal disease), which develops after the arteriovenous (AV) fistula surgery along with the literature review.

## Case presentation

**Figure 1:**
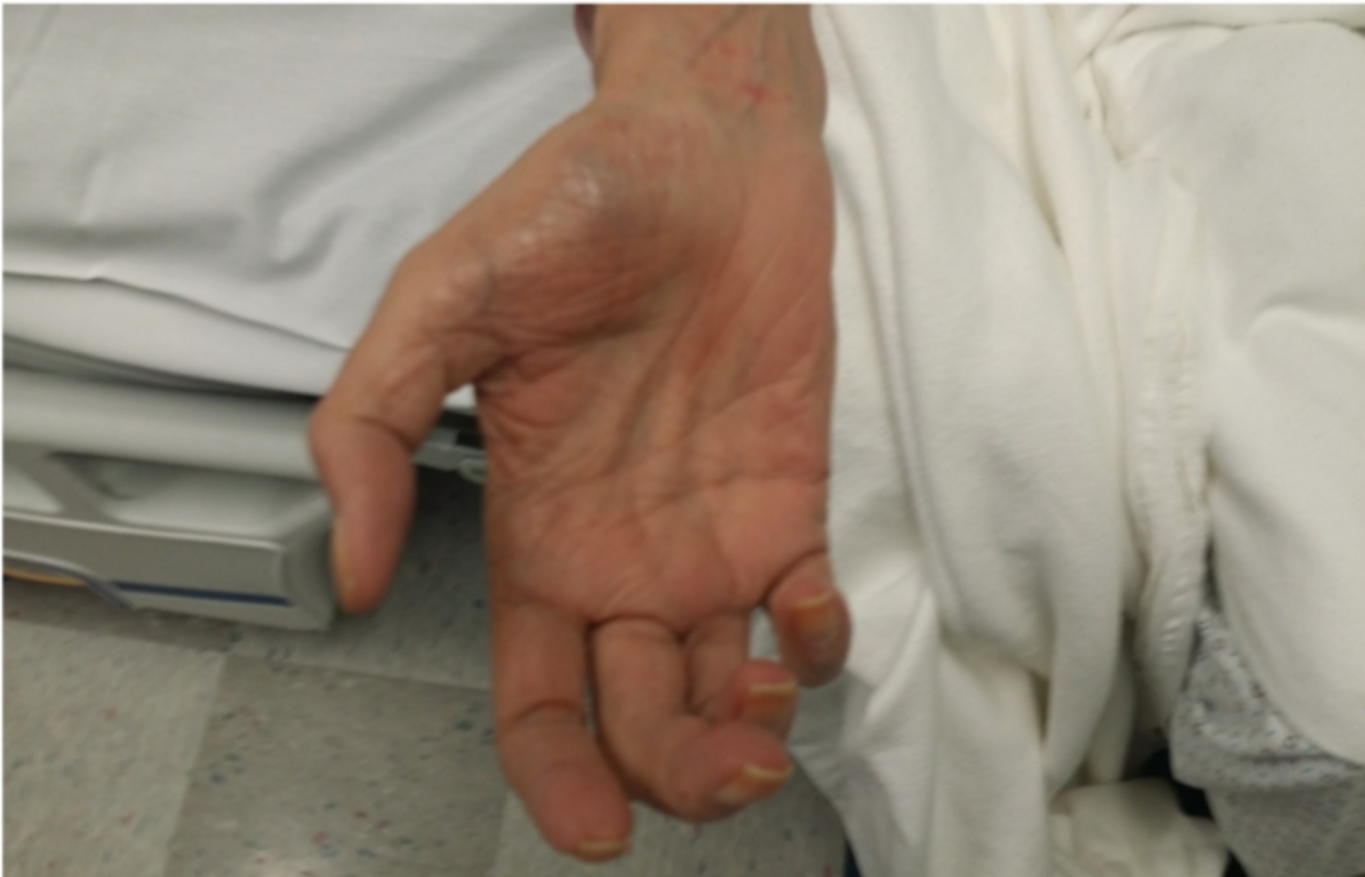
Claw hand due to ischemic monomelic neuropathy

A 70-year-old male patient with the past medical history of end-stage renal disease (ESRD) presented with complaints of right-hand weakness, clumsiness, and pain which developed after one month of brachiocephalic vascular graft surgery. He also experienced a similar kind of a pain in the right hand immediately after the surgery, but it was thought to be secondary to the post-surgical pain at that time. He denied any similar symptoms in the left hand. On the neurological examination, he was noted to have weakness and sensory loss in the right hand, which predominantly involves both median and ulnar nerve distribution (Figure 1).

Nerve conduction studies (NCS) of the right upper extremitity showed reduced motor amplitudes in the median and ulnar nerve. NCS also showed absent sensory amplitudes in the median and ulnar nerves and reduced sensory amplitude in the radial nerve as well. The needle EMG (electromyography) of the right hand showed denervation affecting multiple intrinsic hand muscles supplied by median and ulnar nerves with sparing of more proximal muscles (Table 1). 

**Table 1:** Electromyography summary of the right hand MUAP - Motor Unit Action Potential

Spontaneous							MUAP			Recruitment
		Insertional Activity	Fibrillations	Positive Wave	Fasciculations	Myotonic Discharges	Polyphasia	Amplitude	Duration	Recruit
Right	Extensor digitorum communis	Normal	0	0	0	0	0	Normal	Normal	Full
Right	Deltoid, middle	Normal	0	0	0	0	0	Normal	Normal	Full
Right	Flexor carpi ulnaris	Normal	0	0	0	0	0	Normal	Normal	Full
Right	Flexor pollicis longus	Increased	1	1	0	0	0	Normal	Normal	Full
Right	Pronator teres	Normal	1	1	0	0	0	Normal	Normal	Full
Right	Abductor pollicis previs	Normal	3	3	0	0	0	Normal	Normal	Reduced
Single Motor Unit Potentials Firing										
Right	First dorsal interossei	Normal	2	2	1	0	0	Normal	Normal	Reduced
Right	Biceps brachii	Normal	0	0	0	0	0	Normal	Normal	Full
Right	Triceps	Normal	0	0	0	0	0	Normal	Normal	Full

The Doppler ultrasound studies showed low arterial pressure in the affected hand but with no evidence of vascular steal phenomenon. However, immediately after the vascular access was ligated, his grip strength, involving radial half of digits and thumb was improved, but flexion of the ulnar half of digits continued to show impairment. (Poster: Shah, S, Govindarajan, R. Ischemic Monomelic Neuropathy after Arteriovenous Fistula Surgery: Clinical Features, Electrodiagnostic Findings, and Medicolegal Issues. American Association of Neuromuscular & Electrodiagnostic Medicine Annual Meeting; 09/14/2016).

## Discussion

Patients requiring dialysis with AV fistula vascular graft can potentially be complicated with a rare condition called as ischemic monomelic neuropathy (IMN). IMN was first reported and described in the year 1983 by Wilbourn, et al. [3]. The AV fistula has been preferred over AV graft as vascular access in patients on hemodialysis because of the high patency and also AV fistula has the lowest incidence of mortality and morbidity rate [4-9]. The incidence of IMN is less than one percent of all vascular access procedures [10]. As per the reports from the previous studies, it is suggested that IMN is more likely to be present in females gender and diabetic patients [11, 12].

The pathogenies of IMN is somewhat similar to vascular steal phenomenon, where there is a reduced blood supply to the distal extremity due to the shunting of arterial blood flow into the fistula [13]. Brachiocephalic artery fistulas are at more risk for developing the IMN as the brachial artery is the single artery supplying the forearm and hand. Also, forearm and hand lack the collateral blood supply [2]. Large nerve fibers are supplied with nutrition via vasa nervorum and nutrition to the small nerve fibers is done through localized diffusion from the surrounding. Therefore, we see ischemic damage to the large nerve fibers in IMN [14]. We see symptoms of acute pain, numbness, paresthesia along with the motor weakness. Diffuse mononeuropathy is prominent in these patients in the absence of distal ischemia because the ischemic event in the IMN is brief and immediate [15]. Vascular steal syndrome can be differentiated from the IMN clinically (Table 2).

**Table 2:** Difference between vascular steal syndrome and ischemic monomelic neuropathy

	Vascular steal syndrome	Ischemic monomelic neuropathy
Onset	Insidious	Immediate
Diabetes association	Low association	Very high association
Sex	Variable	Female > Male
Access location	Wrist, forearm, upper arm	Forearm, brachial artery based
Affected tissue	Skin > Muscle > Nerve	Multiple nerves
Clinical ischemia	Severe	Mild
Radial pulse	Absent	Maybe present or absent
Digital pressure	Markedly decreased	Normal or slightly decreased
Reversibility	Variable	Poor

**Figure 2:**
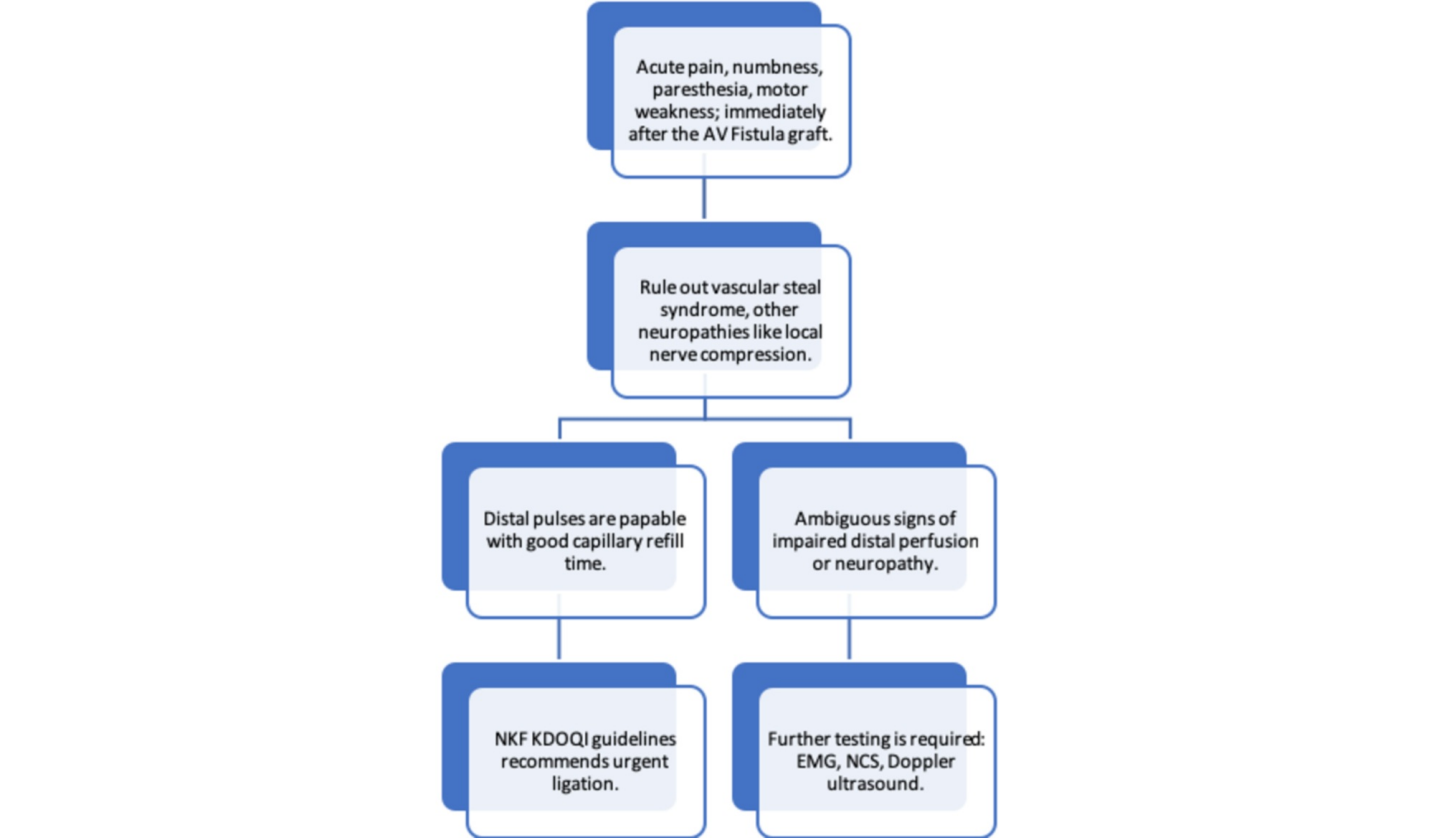
Algorithm showing the diagnosis and management of ischemic monomelic neuropathy AV - arteriovenous; NKF KDOQI - National Kidney Foundation Kidney Disease Outcomes Quality Initiative; EMG - electromyography; NCS - nerve conduction study

Clinical features of distal ischemia, like absent radial pulses, decreased digital arterial pressure, dusky hue discoloration of the fingers, delayed capillary refill time are present more in the vascular steal syndrome as compared to the IMN. IMN is a clinical diagnosis and must be considered especially with post-surgical hand pain. NCS and EMG are useful in the diagnosis, where the former will help in showing diffuse axonal loss along with the reduced nerve conduction velocities of the median, ulnar and radial nerves while EMG shows acute denervation of the upper limb nerves [3]. We can use the algorithm for the diagnosis and management of IMN (Figure 2). 

Despite early recognition and treatment, recovery is incomplete, and patients are left with significant deficits, especially in the first two months after the treatment. This is because of the slow rate of peripheral neuro-regeneration, which is approximately 1mm/day [16]. Clinical studies suggest that early detection and treatment of the IMN will lead to functional recovery of the muscles of the affected limb; therefore, urgent treatment is warranted in these patients [17]. 

## Conclusions

Ischemic monomelic neuropathy is challenging to diagnose because of its initial neurological presentation. Early diagnosis of the condition results in the early closure of the vascular access and can lead to full or partial recovery of the sensory and motor deficits. Any delay in the treatment can lead to irreversible damage.
